# Congenital Midline Cervical Cleft Associated With Transient Antenatal Polyhydramnios Despite Normal Prenatal Imaging: A Case Report

**DOI:** 10.7759/cureus.106777

**Published:** 2026-04-10

**Authors:** Vithura Kunarathnam, Rucha Deshpande, Nathalie Guerrero, Merrai Asad, Izuka Udom-Rice

**Affiliations:** 1 Obstetrics and Gynecology, Nassau University Medical Center, East Meadow, USA; 2 Medicine, American University of the Caribbean School of Medicine, Cupecoy, SXM

**Keywords:** antenatal ultrasound, anterior neck lesion, branchial arch anomaly, cmcc, congenital midline cervical cleft, congenital neck anomaly, infrahyoid lesion, neonatal anomaly, polyhydramnios, prenatal imaging

## Abstract

Congenital midline cervical cleft (CMCC) is a rare congenital anomaly of the anterior neck, typically diagnosed postnatally based on characteristic physical findings. Prenatal detection is unlikely because the lesion is superficial and does not alter fetal anatomy or physiology in a manner detectable on routine imaging. We present a case of CMCC diagnosed after birth in a neonate whose pregnancy was notable only for transient antenatal polyhydramnios. A 27-year-old gravida 2, para 1 presented at term in spontaneous labor following a pregnancy complicated by transient polyhydramnios that resolved prior to delivery. Antenatal evaluation, including ultrasound surveillance and infectious workup, revealed no fetal structural abnormalities. The patient delivered a healthy female neonate via spontaneous vaginal delivery. Postnatal examination identified a midline anterior cervical lesion consisting of a linear cleft with a superior nipple-like projection and an inferior blind-ending sinus tract, consistent with CMCC. Imaging confirmed a superficial infrahyoid lesion without deep extension. This appears to be the first reported case of CMCC associated with antenatal polyhydramnios. This case highlights a limitation of prenatal imaging, as superficial anomalies that do not disrupt fetal physiology may remain undetected despite otherwise normal antenatal evaluation.

## Introduction

Congenital midline cervical cleft (CMCC) is a rare congenital anomaly of the anterior neck, with fewer than 100 cases reported in the literature [1]. CMCC has been reported to occur more frequently in women, with some studies suggesting a female-to-male ratio of approximately 2:1, although data remain limited due to the rarity of the condition [2]. It is typically diagnosed postnatally based on characteristic physical findings. Prenatal detection is unlikely because the lesion is superficial and does not alter fetal anatomy or physiology in a manner detectable on routine imaging. Subtle superficial skin anomalies such as CMCC are therefore difficult to identify prenatally, even when classic features are present at birth.

Prenatal imaging primarily focuses on structural anomalies that affect fetal swallowing, cardiac output, or urinary production, which are the primary contributors to polyhydramnios. As a result, anomalies that do not disrupt these physiologic pathways may remain undetected despite otherwise appropriate antenatal surveillance. This underscores an important limitation of prenatal imaging and highlights the need for careful postnatal examination even when prenatal evaluation appears reassuring.

## Case presentation

A 27-year-old Hispanic G2P1001 at 39 weeks’ gestation by first-trimester ultrasound (estimated due date 12/29/2025; last menstrual period unknown) presented in spontaneous labor. She initially reported intermittent contractions over several days, which increased in frequency and intensity, prompting admission. She denied vaginal bleeding, leakage of fluid, decreased fetal movement, or symptoms of preeclampsia.

On admission, cervical examination revealed 3 cm dilation, 50% effacement, and -3 station. Fetal heart tracing was category I, with contractions every five minutes. Labor progressed spontaneously with the rupture of membranes, and she delivered a viable female neonate via spontaneous vaginal delivery. Apgar scores were 9 and 9 at one and five minutes, respectively, with a birth weight of 3425 g. Delivery was notable for a tight nuchal cord, which was reduced without complication.

The antepartum course was significant for transient polyhydramnios that resolved prior to delivery, preeclampsia without severe features, gestational proteinuria, and herpes simplex virus type 2 seropositivity without clinical outbreaks. The patient received suppressive valacyclovir beginning at 36 weeks’ gestation. There was no personal or family history of congenital anomalies.

First-trimester hemoglobin A1c was 5.4%, and quad screening was negative. A glucose challenge test at 24 weeks and four days was normal at 117 mg/dL. Following the identification of polyhydramnios, repeat glucose testing demonstrated hypoglycemia (52 mg/dL; reference range: 70-130 mg/dL). A growth ultrasound at 34 weeks and three days showed an amniotic fluid index of 28.42 cm, consistent with mild polyhydramnios, prompting referral to high-risk obstetric care. Infectious evaluation revealed elevated cytomegalovirus IgG consistent with prior exposure; all other toxoplasmosis, other agents, rubella, cytomegalovirus, and herpes simplex (TORCH) studies were negative. A follow-up ultrasound at 36 weeks and one day demonstrated the normalization of amniotic fluid (17.45 cm).

The patient’s obstetric history included a prior term vaginal delivery at 38 weeks and four days, complicated by chorioamnionitis and preeclampsia without severe features.

**Figure 1:**
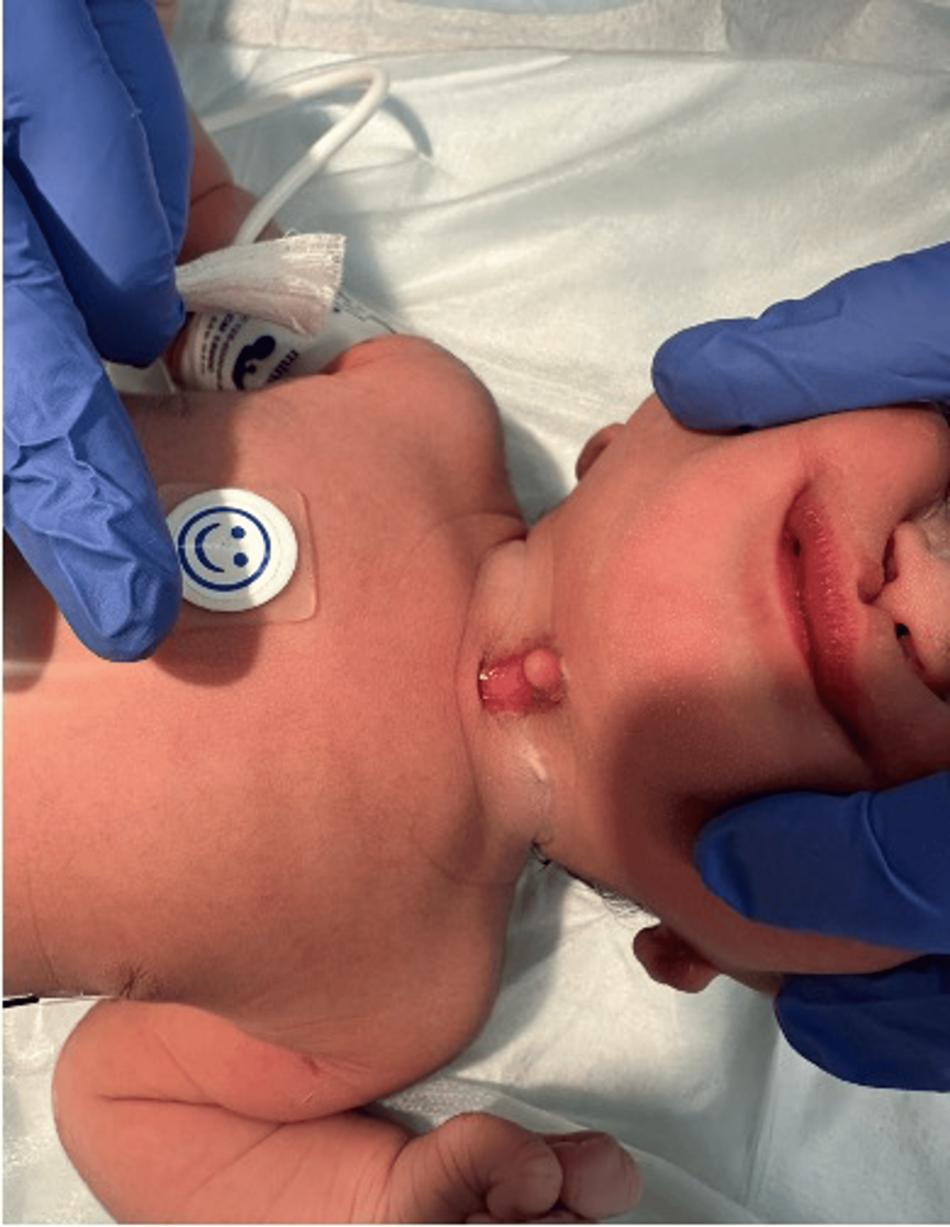
Clinical photograph demonstrating a midline anterior cervical cleft with a superior nipple-like projection and inferior blind-ending sinus tract, consistent with congenital midline cervical cleft.

Postnatal examination revealed a midline anterior cervical lesion extending from the hyoid region to the suprasternal notch, measuring approximately 2-2.5 cm. The lesion consisted of a linear cleft with a superior nipple-like projection and an inferior blind-ending sinus tract, consistent with a congenital midline cervical cleft (Figure 1).

**Figure 2:**
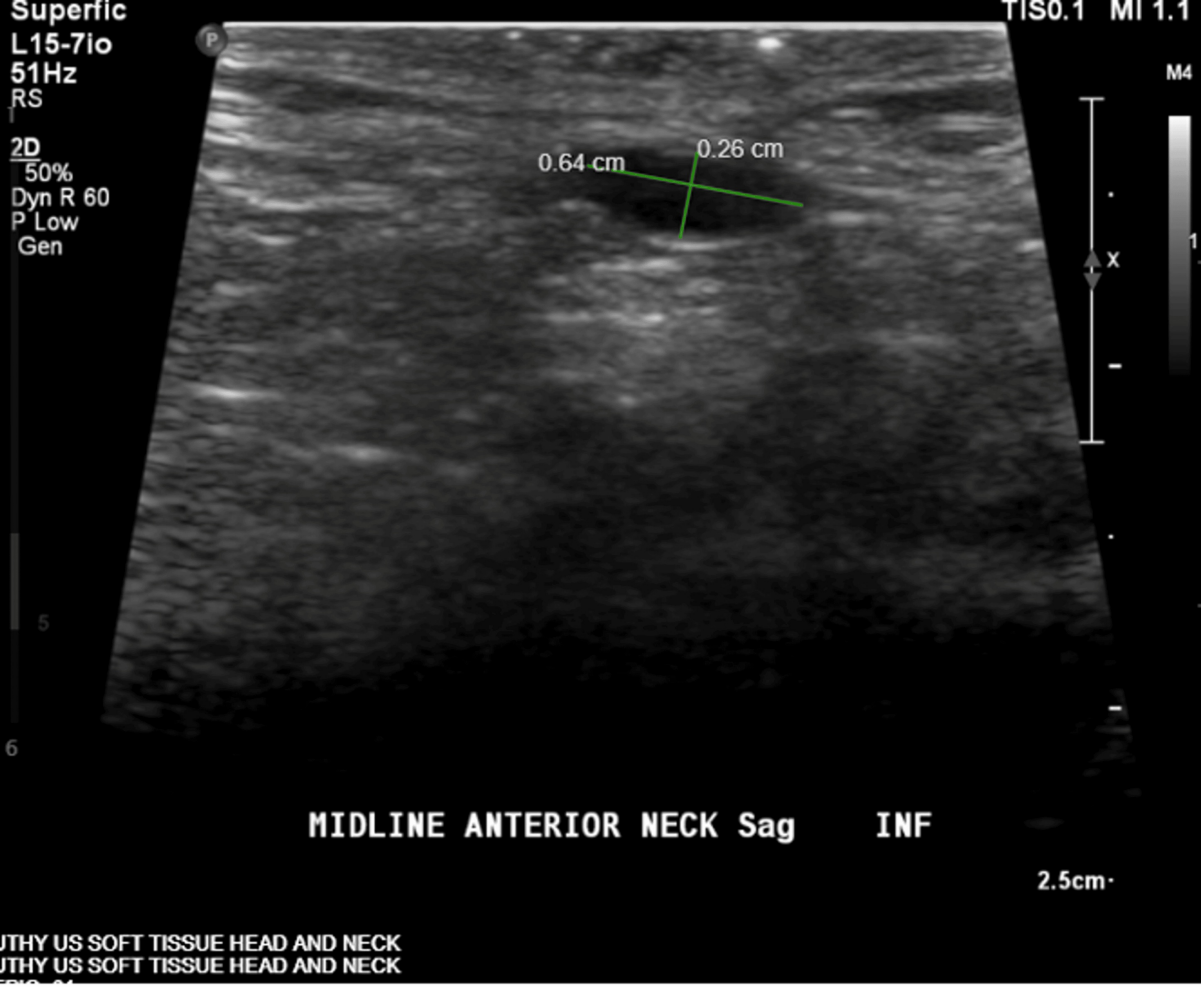
Ultrasound of the anterior neck demonstrating a 0.6 × 0.3 × 0.4 cm cystic lesion within the subcutaneous tissues at the level of the thyroid, with a 0.2 cm hypoechoic focus superiorly and an additional 0.2 cm hypoechoic/cystic area caudally, consistent with congenital midline cervical cleft.

**Figure 3:**
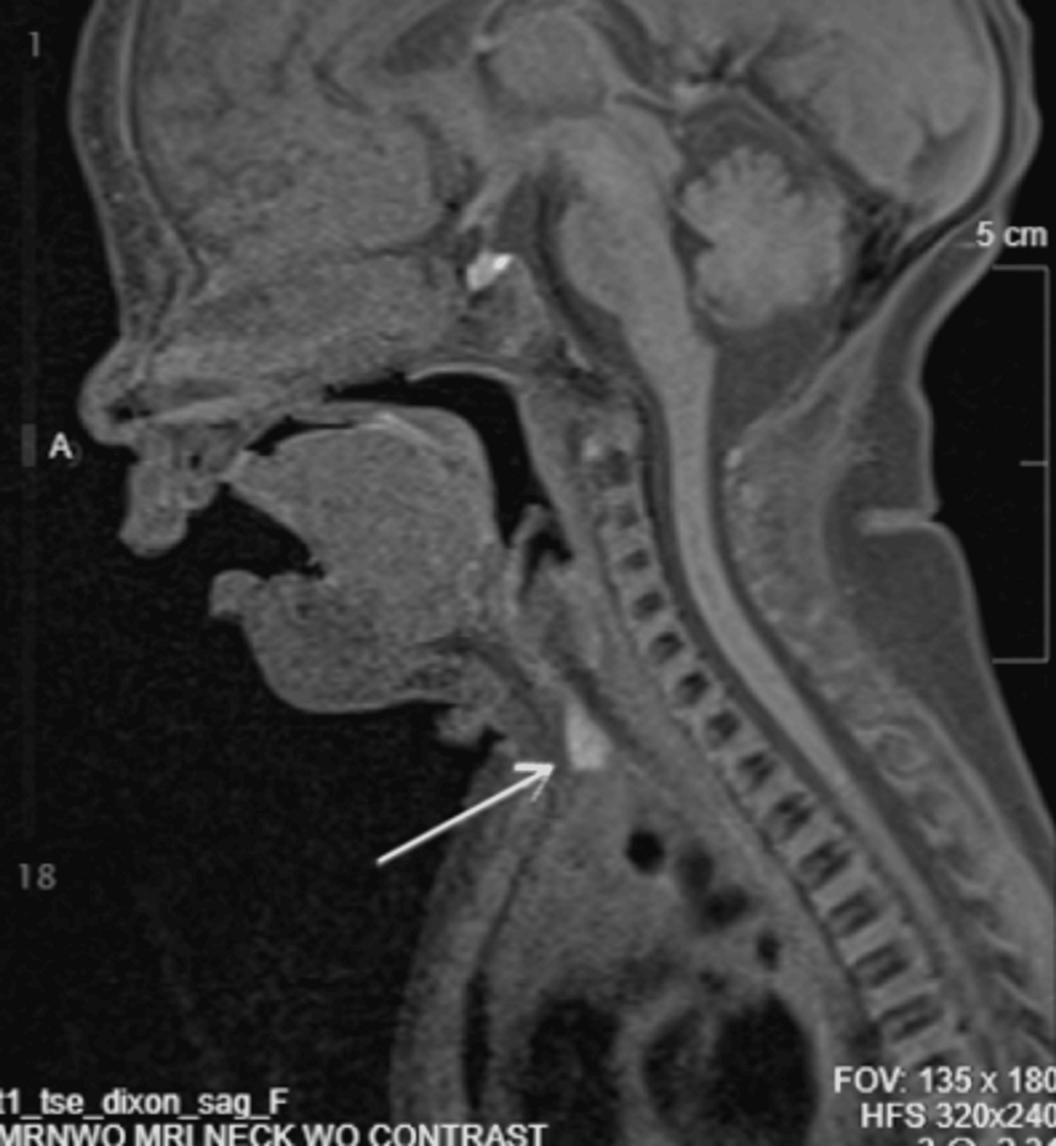
Sagittal MRI of the neck demonstrating a superficial infrahyoid midline lesion (arrow) without evidence of deep extension, consistent with congenital midline cervical cleft. MRI: magnetic resonance imaging

**Figure 4:**
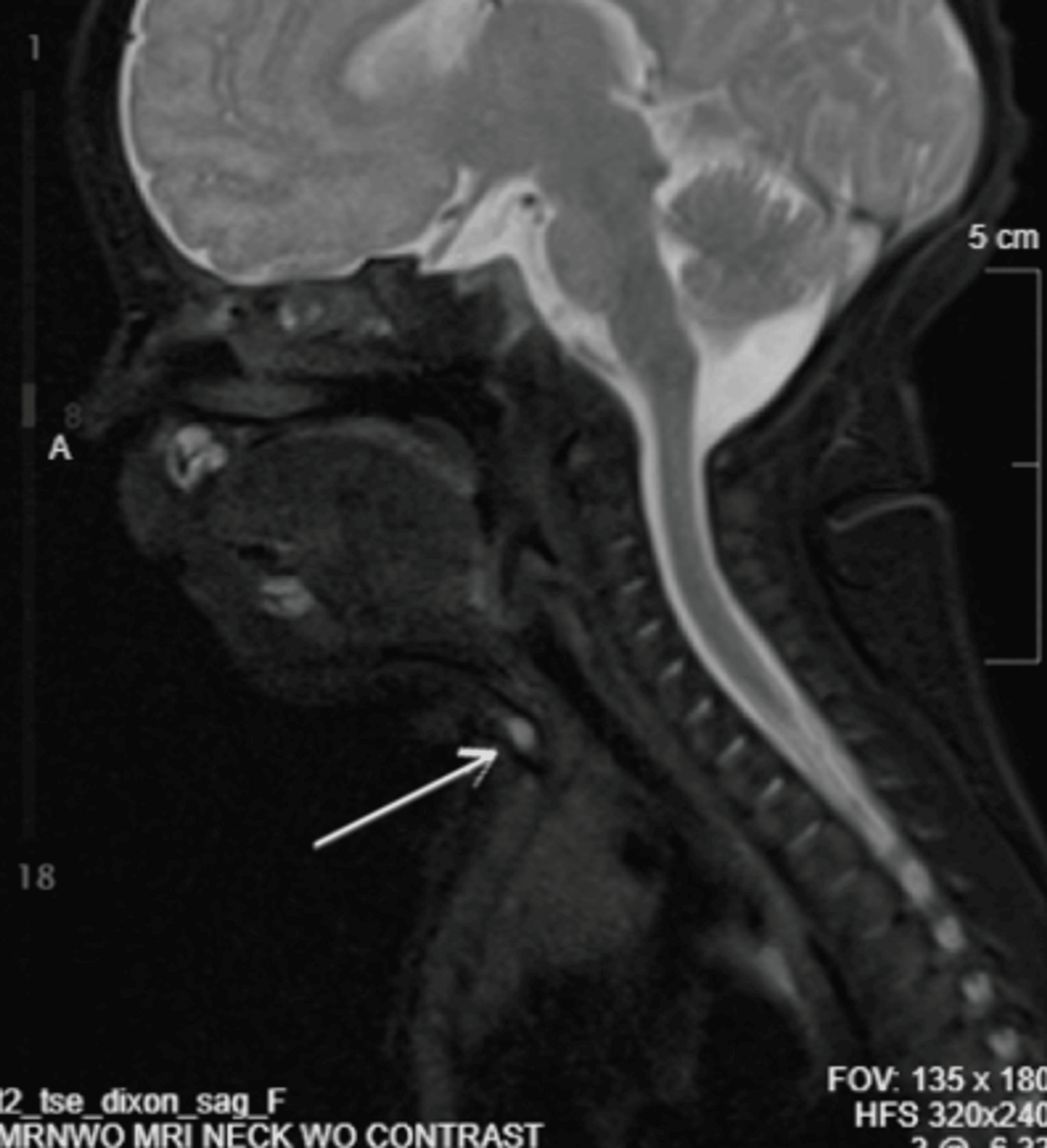
Sagittal MRI of the neck (alternate sequence) demonstrating a superficial infrahyoid midline lesion (arrow) without deep extension, consistent with congenital midline cervical cleft. MRI: magnetic resonance imaging

**Figure 5:**
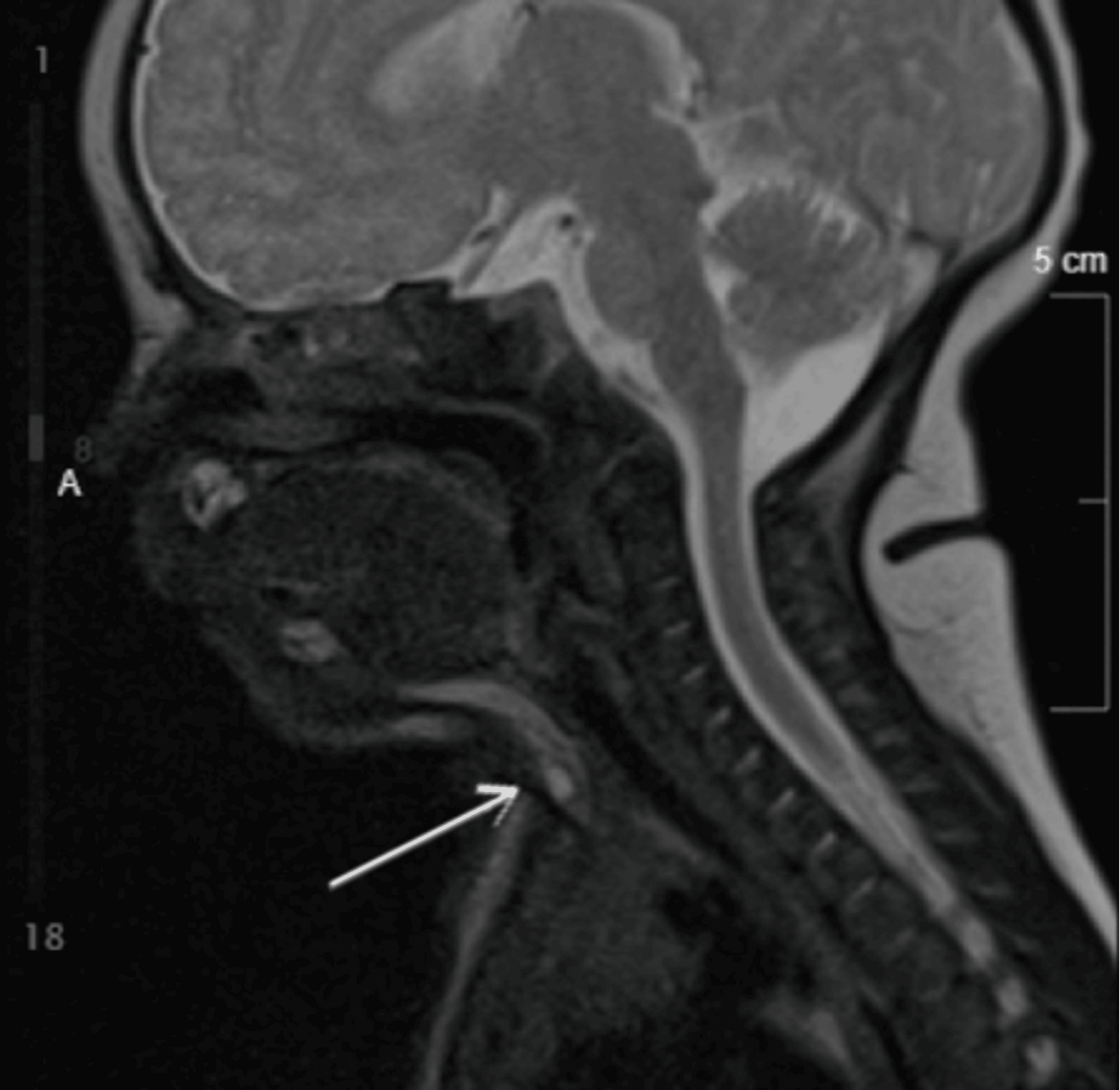
Sagittal MRI of the neck (additional sequence) demonstrating a superficial infrahyoid midline lesion (arrow) without deep extension, consistent with congenital midline cervical cleft. MRI: magnetic resonance imaging

**Figure 6:**
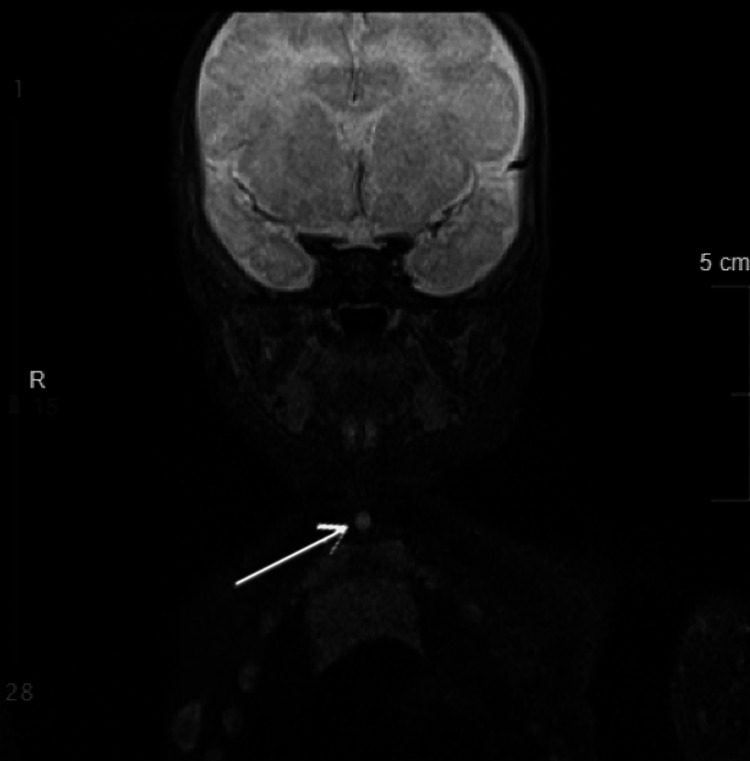
Coronal MRI of the neck demonstrating a midline infrahyoid lesion (arrow) measuring approximately 6 mm, with a small adjacent cystic component (~5 mm), located superficial to the platysma without deep extension, consistent with congenital midline cervical cleft. MRI: magnetic resonance imaging

Ultrasound demonstrated a 0.6 × 0.3 × 0.4 cm cystic lesion within the subcutaneous tissues at the level of the thyroid, with additional smaller hypoechoic areas (Figure 2). Magnetic resonance imaging confirmed a superficial infrahyoid midline lesion without deep extension, supporting the diagnosis (Figures 3-6). Cross-sectional imaging helped differentiate this lesion from other congenital midline neck anomalies, such as thyroglossal duct cysts, dermoid cysts, and branchial cleft anomalies, which typically demonstrate deeper extension, whereas the lesion in this case remained confined to the superficial tissues.

## Discussion

Congenital midline cervical cleft (CMCC) is a rare congenital anomaly of the anterior neck, with fewer than 100 cases reported in the literature [1,3-5]. CMCC has been reported to occur more frequently in women, with some studies suggesting a female-to-male ratio of approximately 2:1, although data remain limited due to the rarity of the condition [2]. It classically presents as a linear midline skin defect associated with an underlying fibrous cord, a superior nipple-like projection, and an inferior blind-ending sinus tract [1,3-5]. The lesion is thought to result from the abnormal fusion of the first and second branchial arches during embryologic development and is typically diagnosed postnatally based on characteristic physical findings [1,3,4].

CMCC has no well-described prenatal ultrasound features. While associated anomalies such as micrognathia may occasionally contribute to polyhydramnios, the superficial cervical defect itself would not be expected to be visualized on routine prenatal imaging. As a result, prenatal detection is highly unlikely even with detailed fetal anatomic surveys [6]. Current fetal imaging is designed to detect anomalies that alter fetal anatomy or physiology, such as those affecting swallowing or amniotic fluid dynamics. In contrast, superficial skin defects that do not disrupt fetal function may fall outside the resolution of routine prenatal evaluation.

The lesion in this case demonstrated all three classic components of CMCC, a combination reported in a minority of cases [3,4]. Cross-sectional imaging was useful in distinguishing this lesion from other congenital midline neck anomalies, including thyroglossal duct cysts, dermoid cysts, and branchial cleft anomalies, which typically demonstrate deeper extension [1]. The superficial location observed in this case is consistent with the classic triad described in the literature [1,3,4].

The transient polyhydramnios observed in this pregnancy is most consistent with idiopathic polyhydramnios. Idiopathic polyhydramnios is a diagnosis of exclusion, as underlying fetal anomalies may only become apparent after birth. Studies suggest that 9%-28% of cases initially classified as idiopathic are later associated with structural or genetic abnormalities diagnosed postnatally [7,8]. One study of 134 children with polyhydramnios and otherwise normal prenatal evaluation found that 19% were subsequently diagnosed with congenital malformations, with increased rates of genetic syndromes and neurologic disorders compared to controls [7]. Idiopathic polyhydramnios accounts for approximately 50%-60% of cases and may resolve spontaneously [9].

Polyhydramnios complicates approximately 1%-2% of pregnancies and is most often mild and idiopathic [9,10]. When identified, evaluation appropriately focuses on conditions that impair fetal swallowing or increase fetal urine production [11]. However, as demonstrated in this case, anomalies that do not disrupt these physiologic pathways may remain undetected despite appropriate antenatal surveillance.

From a clinical perspective, this case has implications for prenatal counseling. Although patients with idiopathic or transient polyhydramnios are often reassured when fetal imaging is normal, rare structural anomalies may still only become apparent after birth. Careful neonatal examination therefore remains essential even when prenatal evaluation appears reassuring.

This appears to be the first reported case of CMCC associated with antenatal polyhydramnios. CMCC itself would not be expected to directly cause polyhydramnios, as the lesion is superficial and does not interfere with fetal swallowing or amniotic fluid regulation. Although CMCC may initially appear to be a superficial or cosmetic anomaly, untreated lesions can result in functional complications, including cervical contracture, restricted neck extension, and progressive deformity due to the presence of an underlying fibrous cord [12]. The polyhydramnios in this case likely represented an incidental finding rather than a direct physiologic consequence of the cervical cleft. Early surgical excision with reconstruction, typically using Z-plasty techniques, is the recommended treatment and is ideally performed in infancy, often within the first year of life, to prevent the development of contracture and optimize functional and cosmetic outcomes [3,5,13]. This case underscores the importance of maintaining clinical vigilance in pregnancies complicated by idiopathic or transient polyhydramnios, as rare congenital anomalies may only become apparent after birth despite otherwise reassuring prenatal imaging.

## Conclusions

The inability to detect CMCC prenatally reflects inherent technical limitations of current imaging modalities rather than inadequate screening. Routine fetal surveys prioritize the detection of structural abnormalities affecting swallowing, cardiac output, or urinary production, which are the primary contributors to polyhydramnios. Subtle superficial cervical skin defects fall outside the scope of typical prenatal evaluation and are unlikely to be detected even with advanced imaging.

This case highlights the gap between prenatal imaging capabilities and postnatal findings. The absence of detectable fetal anomalies does not exclude the possibility of rare structural conditions that may only become evident after delivery. Careful neonatal examination therefore remains essential, even when prenatal evaluation appears reassuring.
